# Impact of Airstacking and Digital Pressure Feedback on Pulmonary Function in Restrictive Lung Disease: A Stratified Randomized Controlled Trial

**DOI:** 10.3390/biomedicines13030616

**Published:** 2025-03-03

**Authors:** Han Eol Cho, Won Ah Choi, Seul Lee, Seong-Woong Kang

**Affiliations:** 1 Department of Rehabilitation Medicine, Gangnam Severance Hospital, Yonsei University College of Medicine, Seoul 06273, Republic of Korea; wtzephyr@yuhs.ac (H.E.C.); reedlove37@yuhs.ac (W.A.C.);; 2 Rehabilitation Institute of Neuromuscular Disease, Yonsei University College of Medicine, Seoul 03722, Republic of Korea

**Keywords:** airstacking, neuromuscular disorders, pulmonary rehabilitation, digital feedback, caregiver outcomes

## Abstract

**Background/Objectives:** Airstacking is a technique to improve lung compliance and maximum insufflation capacity (MIC) in patients with neuromuscular disorders by sequentially inflating the lungs using a manual resuscitation bag. Traditional methods lack standardization and rely on subjective feedback. A pilot study established optimal pressure ranges using a digital manometer, suggesting its potential to standardize airstacking. This study evaluates the longitudinal effects of airstacking with and without digital pressure feedback on pulmonary function. **Methods:** A stratified randomized controlled trial was conducted with 40 patients allocated into three groups: those performing airstacking appropriately (Group 1), those previously performing airstacking inappropriately but using digital pressure feedback during the study (Group 2), and those previously performing airstacking inappropriately without feedback (Group 3). Pulmonary function parameters, including forced vital capacity expressed as a percentage of the predicted normal value (FVC%), MIC, and assisted peak cough flow (aPCF), were measured at baseline, 3, 6, and 12 months. Caregiver outcomes, musculoskeletal pain, and satisfaction were assessed. **Results:** Digital pressure feedback did not significantly alter pulmonary function. Changes in FVC% (*p* = 0.164), MIC (*p* = 0.218) and aPCF (*p* = 0.787) were not statistically significant. However, Group 2 caregivers showed significant reductions in musculoskeletal pain than Group 3 (*p* = 0.036) and higher satisfaction (mean: 8.92/10). The proportion of caregivers achieving optimal pressure increased by 25% in Group 2 compared to 16.67% in Group 3. **Conclusions:** While digital pressure feedback did not significantly alter pulmonary function, it contributed to improved caregiver adherence and reduced musculoskeletal pain. These findings suggest that integrating objective pressure feedback into airstacking training may enhance technique standardization and caregiver experience, though its impact on pulmonary function remains uncertain.

## 1. Introduction

Patients with neuromuscular disorders experience the progressive weakening of respiratory muscles. This leads not only to a reduction in forced vital capacity (FVC) but also to a decrease in chest wall compliance, which subsequently reduces maximum insufflation capacity (MIC) [1,2]. The decline in MIC ultimately diminishes cough flow [2] and predisposes patients to pulmonary complications such as atelectasis and pneumonia. Maintaining MIC has been identified as a critical factor for preventing these complications even when the decline in FVC is inevitable.

Airstacking has been widely adopted as an effective intervention to enhance lung compliance and MIC in patients with neuromuscular disorders [3,4,5,6,7]. By using a manual resuscitation bag, airstacking helps to improve lung and chest wall compliance, enabling patients to achieve and maintain maximum insufflation capacity [8]. The exercise involves sequentially inflating the lungs by stacking volumes of air through multiple breaths without exhalation. This process is facilitated by using the manual resuscitation bag to deliver additional air after the patient has inhaled fully. Careful coordination between the patient and caregiver ensures that the air is stacked effectively without causing discomfort. Studies have demonstrated its potential to improve cough flow and maintain pulmonary health [2,4,9]. However, the subjective nature of traditional methods, which rely on the patient’s perception and the caregiver’s tactile feedback, limits their reproducibility and effectiveness. Prior studies have primarily relied on subjective feedback without standardized monitoring tools, limiting reproducibility. Although our pilot study [8] established optimal pressure ranges, most prior research lacked such standardized evaluation methods, making it difficult to assess airstacking outcomes consistently.

In our prior pilot study [8], we objectively identified the peak pressure during airstacking performed by experts and established optimal pressure ranges. These findings suggested that a digital manometer could significantly aid patients and caregivers in accurately performing airstacking, offering a standardized approach to this critical therapy. However, the previous study was limited to cross-sectional analyses and did not explore the longitudinal effects of standardized airstacking practices.

Building on these findings, this study aims to evaluate the effectiveness of a standardized airstacking technique with and without the use of digital pressure feedback on pulmonary function in patients with neuromuscular disorders. By assessing key pulmonary function parameters over time, we try to provide comprehensive evidence of the clinical benefits of standardized airstacking techniques. The results will contribute to improving pulmonary care protocols and optimizing long-term outcomes for patients with neuromuscular disorders.

## 2. Materials and Methods

### 2.1. Participants

This study included individuals with neuromuscular disorders who visited the Pulmonary Rehabilitation Center of a tertiary hospital in South Korea between 2022 and 2023. Eligible participants were 13 years or older, had respiratory muscle weakness, and demonstrated a vital capacity less than 50% of the predicted value. To minimize variability in prior airstacking practice, only individuals who had been regularly performing airstacking after receiving training at the center were included.

The exclusion criteria were patients with tracheostomy, a history of pneumothorax or other conditions increasing the risk of airstacking, significant bulbar dysfunction affecting airstacking performance, cognitive impairments preventing cooperation, or unwillingness to participate in the study. Dropout was defined as participants who did not complete the study, with reasons including the withdrawal of consent, violation of the inclusion/exclusion criteria, serious adverse events or adverse events, inability to follow up, or other factors deemed appropriate by the investigators.

#### Sample Size Calculations

The target sample size was calculated using G*Power (version 3.1.9.7) to ensure adequate statistical power for detecting within–between interaction effects in a repeated measures ANOVA design. An effect size of 0.25 (moderate effect) was chosen based on statistical and practical considerations rather than being directly derived from prior studies. While previous research has examined airstacking and pulmonary rehabilitation in neuromuscular patients, variability in study designs and outcome measures made it difficult to determine a single definitive effect size applicable to our study. Our pilot study [8] provided preliminary insights into the impact of pressure feedback, but the sample size was insufficient for precise effect size estimation. Given the need to balance statistical sensitivity with study feasibility, we selected an effect size of 0.25 as a reasonable assumption for detecting clinically meaningful differences.

Using this effect size, 3 groups, 4 repeated measurements, a correlation among repeated measures of 0.5, and a nonsphericity correction factor of 1.0, the required total sample size was determined to be 33 participants. This calculation was based on an alpha level of 0.05 and a statistical power of 85% (actual power = 0.85). To account for a potential dropout rate of 15%, the target sample size was adjusted to 39 participants. However, to ensure robustness against unforeseen circumstances, a total of 40 participants were recruited, ensuring sufficient data would be available to maintain statistical rigor despite anticipated attrition.

### 2.2. Study Design

This study was designed as a single randomized controlled study to evaluate the effects of airstacking on pulmonary function in individuals with neuromuscular disorders. A stratified and partial randomization approach was employed to allocate the participants into three groups. The participants were first stratified based on their appropriateness for performing airstacking, which was determined by two experts based on the criteria established in prior research [8]. Appropriateness was assessed using three key criteria: (1) synchronization between caregiver-assisted bag compression and the participant’s inhalation, (2) the participant’s perception of chest fullness, (3) the alignment of the participant’s chest fullness sensation with the caregiver’s perception of resistance during bag compression, and (4) peak inspiratory pressure exceeding 35 cmH_2_O. If all the criteria were met, the participant was assigned to the appropriate force group (Group 1). If any criterion was not met, the participant was considered inappropriate for airstacking and was randomized into Group 2 (digital pressure feedback) or Group 3 (no feedback). Group 2 was provided with a digital manometer to maintain a target peak inspiratory pressure over 35 cmH_2_O based on prior studies demonstrating optimal peak pressure of airstacking. By using a digital manometer, the participants received real-time feedback to guide their effort and adjust their technique accordingly. All the participants performed 15 maneuvers of airstacking twice a day [7].

### 2.3. Intervention and Outcome Measures

Before the study began, the clinical and spirometric data of the participants were collected, including age, sex, diagnosis, FVC, MIC, and assisted peak cough flow (aPCF). Additionally, muscular–skeletal pain among the caregivers who performed the airstacking was assessed. After the initial evaluation, a specialist in pulmonary rehabilitation for patients with neuromuscular disorders with over 10 years of experience provided direct training on how to perform airstacking. The participants randomized to Group 2 received digital manometers and were trained in their use. The study employed a repeated measures design, with the assessments conducted at baseline, 3 months, 6 months, and 12 months. FVC was measured using a portable spirometer (Micro Medical Ltd., Rochester, Kent, UK) with the participants in a sitting position [10] and expressed as a percentage of the predicted normal value (FVC%) [11]. MIC was attained by the participant taking a deep breath and holding it; airstacking was then used to consecutively deliver volumes of air via an oral–nasal interface. aPCF was measured by applying an abdominal thrust during MIC to record the maximum flow [10]. Musculoskeletal pain among the caregivers who performed the airstacking e was assessed using a visual analog scale (VAS) to quantify pain levels around the wrist and hand. Additionally, satisfaction with the digital manometer was assessed in Group 2 using a 0 to 10 score.

### 2.4. Blinding of Outcome Assessors

Outcome assessors were blinded to group allocation to ensure unbiased data collection and analysis, thereby enhancing the reliability of measured outcomes

### 2.5. Statistical Analysis

All the statistical analyses were performed using R version 3.6.1. Descriptive statistics were used to summarize the demographic and baseline clinical characteristics of the participants. Continuous variables were expressed as mean ± standard deviation (SD).

For the primary outcomes (FVC, MIC, and aPCF), a repeated measures ANOVA was conducted to assess within-group changes and between-group differences over time (baseline, 3, 6, and 12 months). Post hoc comparisons were performed using Tukey’s test to identify specific group differences. Statistical significance was set at *p* < 0.05.

## 3. Results

### 3.1. Basic Characteristics of Patients

A total of 40 patients were initially recruited for the study with a mean age of 26.6 ± 6.8 years. Among them, 33 had Duchenne muscular dystrophy, 5 had spinal muscular atrophy, 1 had myotonic muscular dystrophy, and 1 had hereditary myopathy. The cohort included 37 male and 3 female participants.

**Figure 1:**
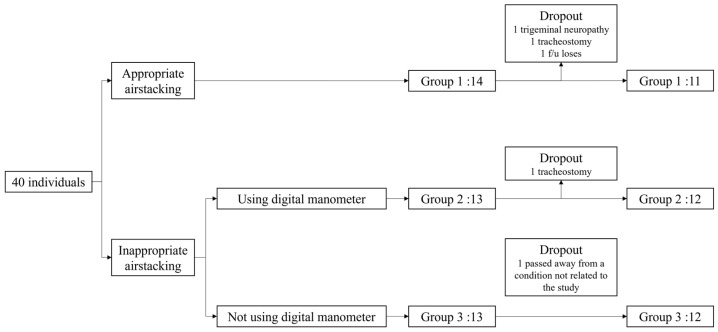
Flowchart of participant allocation, exclusions, and study completion.

The patients were stratified into three groups: 14 in Group 1, 13 in Group 2, and 13 in Group 3. Among them, 11 patients in Group 1 and 12 patients in each of Groups 2 and 3 completed the study, resulting in a final cohort of 35 participants. The reasons for dropout included the following: in Group 1, three participants were excluded—one due to trigeminal neuralgia preventing airstacking, one due to undergoing tracheostomy, and the other due to follow-up loss. In Group 2, one participant dropped out due to a tracheostomy. In Group 3, one participant was excluded due to passing away from a condition not related to the study (Figure 1).

Group 1 has a mean age of 27.8 ± 7.0 years, Group 2 having 25.3 ± 6.8 years, and Group 3 having 25.0 ± 6.9 years. A one-way ANOVA revealed no statistically significant difference in age between the three groups (*p* = 0.573), indicating comparability in age distribution.

The distribution of diagnoses across the three groups was also analyzed. Group 1 included nine patients with Duchenne muscular dystrophy and two with spinal muscular atrophy. Group 2 included 10 patients with Duchenne muscular dystrophy, 1 with spinal muscular atrophy, and 1 with congenital myopathy. Group 3 included nine patients with Duchenne muscular dystrophy, two with spinal muscular atrophy, and one with myotonic muscular dystrophy. A chi-square test revealed no statistically significant differences in diagnosis distribution among the groups (χ^2^ = 4.35, *p* = 0.629).

### 3.2. Analysis of Forced Vital Capacity Changes

Baseline FVC values were comparable among the groups. The mean ± SD FVC (mL) at baseline was 564.55 ± 362.91 for Group 1, 935.00 ± 521.88 for Group 2, and 920.00 ± 532.05 for Group 3. For analysis, FVC% was used as described in the methods section.

**Figure 2:**
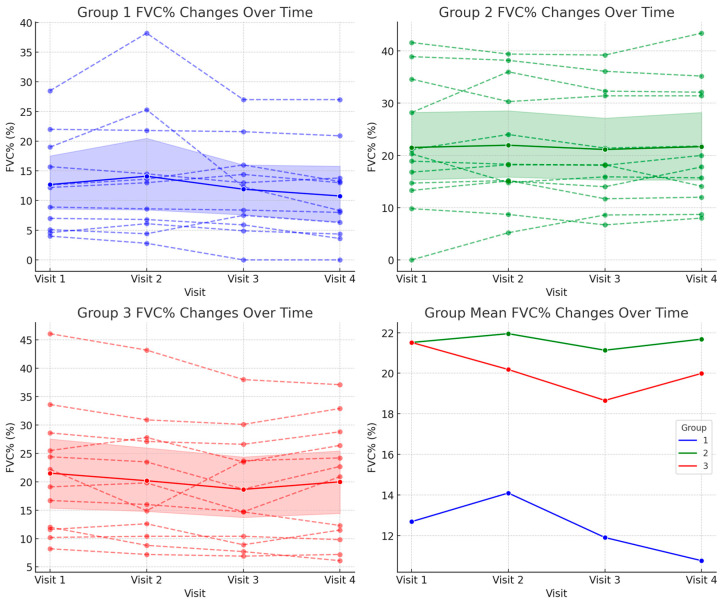
Changes in forced vital capacity (FVC%) over 12 months across the three groups.

The mean FVC% values and standard deviations for each group at baseline (Visit 1) and at 12 months (Visit 4) are summarized as follows: Group 1 showed a decrease from 12.69 ± 7.94% at baseline to 10.77 ± 7.93% at 12 months. In contrast, Group 2 remained relatively stable, with values of 21.95 ± 12.81% and 22.37 ± 11.64% at baseline and 12 months, respectively. Similarly, Group 3 showed minimal change, decreasing slightly from 21.95 ± 11.50% to 20.69 ± 10.64% (Figure 2 and Table 1).

The repeated measures ANOVA revealed no significant interaction effects between the group and time (*p* = 0.164), indicating that FVC% changes were not statistically significant over the 12-month period, nor did they differ among the groups.

These findings suggest that over the course of the 1-year study period, the rate of change in FVC% was similar across all the groups, and the observed differences in FVC% between the groups did not reach statistical significance.

### 3.3. Changes in MIC and aPCF

We assessed the changes in MIC and aPCF across three groups over a 12-month period using repeated measures ANOVA. The MIC measurements were taken at four distinct time points: Visit 1 (baseline), Visit 2, Visit 3, and Visit 4.

**Figure 3:**
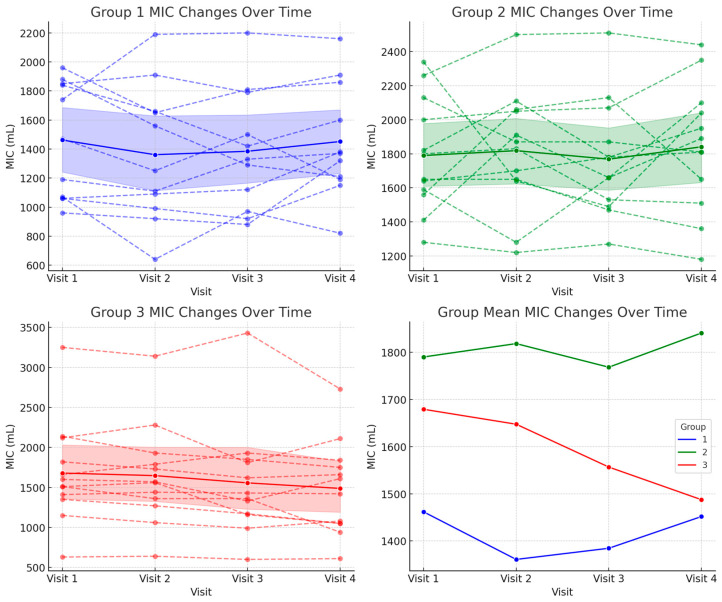
Changes in maximum insufflation capacity (MIC) over 12 months across the three groups.

For MIC, Group 1 showed a decrease in MIC from 1461.82 ± 399.55 mL at baseline to 1451.82 ± 393.26 mL at Visit 4. Group 2, on the other hand, remained relatively stable, with MIC values of 1790.00 ± 333.52 mL at baseline and 1840.83 ± 375.99 mL at 12 months. Group 3 showed minimal change, decreasing slightly from 1679.17 ± 640.99 mL to 1487.50 ± 587.17 mL (Figure 3 and Table 2).

**Figure 4:**
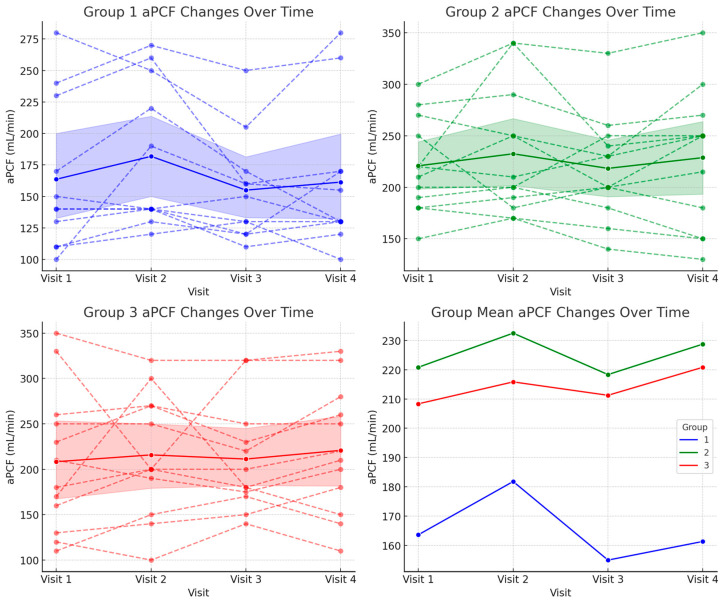
Changes in assisted peak cough flow (aPCF) over 12 months across the three groups.

For aPCF, Group 1 showed a decrease in aPCF from 1461.82 ± 399.55 mL/min at baseline to 1451.82 ± 393.26 mL/min at Visit 4. Group 2, on the other hand, remained relatively stable, with aPCF values of 1790.00 ± 333.52 mL/min at baseline and 1840.83 ± 375.99 mL/min at 12 months. Group 3 showed minimal change, decreasing slightly from 1679.17 ± 640.9 mL/min 9 at baseline to 1487.50 ± 587.17 mL/min at Visit 4 (Figure 4 and Table 3).

Although the mean MIC and aPCF values decreased in Group 1 and Group 3 and increased in Group 2, the repeated measures ANOVA analysis revealed no significant interaction effects between group and time (*p* = 0.164 for MIC and *p* = 0.787 for aPCF), suggesting that the MIC and aPCF changes were not statistically significant over the 12-month period. These findings suggest that the rate of change in MIC and aPCF was similar across all the groups, and the observed differences did not reach statistical significance over the 12-month period.

### 3.4. Caregiver Outcomes: Efficacy, Pain, and Satisfaction with Digital Manometry

The proportion of caregivers achieving a peak pressure ≥35 increased from Visit 1 to Visit 4 across all the groups. In Group 1, the proportion remained stable, with 9 out of 11 caregivers meeting the threshold at both visits (81.82%). Group 2 demonstrated a notable improvement, increasing from 7 out of 12 caregivers (58.33%) at Visit 1 to 10 out of 12 caregivers (83.33%) at Visit 4, representing a 25.00% improvement. Similarly, Group 3 increased from 6 out of 12 caregivers (50.00%) to 8 out of 12 caregivers (66.67%), with a 16.67% improvement.

**Figure 5:**
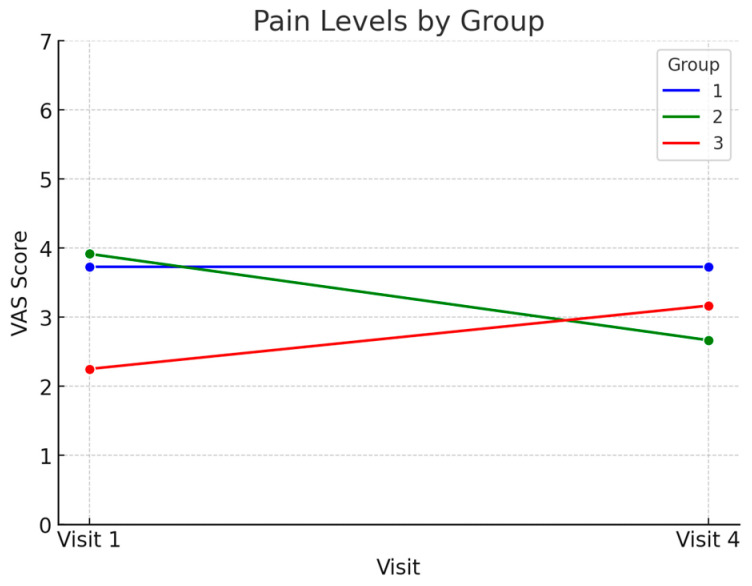
Changes in caregiver musculoskeletal pain (VAS scores) from baseline to 12 months.

The changes in musculoskeletal pain were evaluated using a VAS at baseline (Visit 1) and the follow-up (Visit 4). Group 1 showed no significant change in pain levels, maintaining a mean score of 3.73 ± 2.94 across both time points, and Group 3 showed a slight increase in pain levels from 2.25 ± 3.08 at Visit 1 to 3.17 ± 2.66 at Visit 4. In contrast, Group 2 exhibited a reduction in pain levels, with the scores decreasing from 3.92 ± 3.15 at Visit 1 to 2.67 ± 2.67. The repeated measures ANOVA revealed a significant interaction effect between group and time (*p* = 0.045), indicating that the trajectory of pain levels differed among the groups. Post hoc analyses confirmed that the pain reduction observed in Group 2 was significantly greater than in Group 3 (*p* = 0.036), while no significant difference was observed between Groups 1 and 2 or Groups 1 and 3 (*p* > 0.05) (Figure 5).

In addition to pain reduction, caregivers in Group 2 reported high satisfaction with the use of the digital manometer. The average satisfaction score at Visit 4 was 8.92 per 10, suggesting that the device was well received and contributed to a more efficient and comfortable airstacking experience. These findings highlight the potential utility of digital pressure feedback devices in improving not only the physical burden of caregiving but also the overall satisfaction associated with the intervention.

## 4. Discussion

Airstacking is a vital pulmonary rehabilitation technique for patients with neuromuscular disorders [12], designed to maintain lung and chest wall compliance while enhancing MIC. The progressive weakening of respiratory muscles in these patients inevitably increases the risk of pulmonary complications, such as atelectasis and pneumonia, which significantly affect both the quality of life and survival. Thus, airstacking serves as an essential intervention to prevent these complications by supporting MIC maintenance and improving effective coughing [13,14] and ventilation [15,16].

Traditionally, airstacking has relied heavily on the subjective experience of patients and caregivers. However, by providing a more reliable method to achieve optimal insufflation, this approach holds significant potential for improving the pulmonary rehabilitation protocols for patients with neuromuscular disorders [17]. In our prior pilot study, we identified and proposed optimal pressure ranges to quantify airstacking, addressing the need for standardization [8]. Building on this foundation, the present study aimed to evaluate the effectiveness of digital pressure feedback with a cheap and small manometer in enhancing airstacking practices and its impact on pulmonary function in this patient population.

Over the 1-year study period, no statistically significant differences were observed in the changes in FVC, MIC, or aPCF among the three groups. This indicates that the use of a digital manometer did not provide additional benefits in improving pulmonary function compared to the conventional airstacking methods. Specifically, our study did not observe significant changes in MIC over time, whereas previous studies [2,5] have reported a gradual increase in MIC with continued airstacking practice. We believe the primary reason for this discrepancy is that all the participants had prior experience with airstacking before the study. Only patients from our outpatient clinic who were already familiar with airstacking were included, and those new to the technique were excluded. Furthermore, all the participants received standardized training at the beginning of the study, which likely optimized their baseline performance, leaving little room for further measurable improvement. While digital feedback provided a more intuitive understanding of the process, the participants without the feedback device still benefited from the standardized training, potentially diminishing the observable impact of digital feedback on pulmonary function outcomes.

A notable observation in this study is the trend toward a reduced proportion of caregivers performing airstacking at suboptimal pressure levels, which is often a challenge in effectively implementing this technique. Group 2, which utilized digital pressure feedback, showed an improvement in achieving appropriate pressure levels during airstacking. Specifically, the proportion of caregivers reaching the target peak pressure (≥35 cmH_2_O) increased from 58.33% at Visit 1 to 83.33% at Visit 4, representing a 25.00% improvement. In comparison, Group 3, which did not use digital feedback, demonstrated a 16.67% improvement, increasing from 50.00% to 66.67%. These findings suggest that digital feedback devices may help address the challenge of insufficient pressure application during airstacking, which can limit its effectiveness. The use of manometers provided real-time feedback, potentially enabling caregivers to apply adequate pressure more consistently compared to those relying on manual estimation. This suggests that incorporating objective tools such as manometers into airstacking protocols could be beneficial, particularly for caregivers with varying levels of experience or confidence in their technique.

Also, an interesting result of this study relates to the musculoskeletal pain experienced by caregivers [18,19]. The caregivers in Group 2 reported a clear reduction in pain compared to the other groups. In our prior pilot study, nearly 70% of the caregivers reported chronic wrist and hand pain associated with performing airstacking regularly. Digital feedback devices likely reduced the need for excessive force during airstacking, offering caregivers an opportunity to alleviate chronic pain. This highlights an important secondary benefit of digital feedback devices not only for patients but also for the well-being of their caregivers.

In line with a previous study [8], airstacking can be challenging for caregivers without clear guidance on how to determine the endpoint of each maneuver. This often leads to either insufficient or excessive air delivery. Caregivers may feel pressured to apply excessive force or perform the technique for longer durations, believing that stronger efforts are more effective, or they may deliver too little air due to concerns about patient safety. The use of a digital manometer provided real-time feedback, allowing the caregivers to maintain air delivery within the optimal range. This improved force control and precision, reducing uncertainty and physical strain while preventing both under- and over-delivery of air.

These results may partly explain the high level of satisfaction reported by the caregivers regarding the use of the digital manometer. The caregivers who used the digital manometer rated their experience with the device at an average of 8.92 out of 10, reflecting a high level of satisfaction. This suggests that the device not only enhanced their confidence in applying the correct pressure during airstacking but also made the process more manageable and less physically demanding. Positive feedback further supports the practicality of integrating digital feedback into routine airstacking protocols, especially in settings where caregiver fatigue and burden are significant concerns.

Furthermore, the high satisfaction score indicates a strong willingness among the caregivers to continue using the device in the long term. This is particularly relevant for patients requiring lifelong respiratory support, as the sustained use of such devices could contribute to better adherence to airstacking protocols and potentially improve long-term outcomes for patients. By simplifying the process and reducing physical strain, digital feedback devices appear to provide an important solution to the challenges faced by caregivers, thereby ensuring more consistent and effective care for patients.

We utilized an inexpensive, small, and lightweight portable digital manometer to ensure accessibility for patients and caregivers. This approach aimed to lower the barriers to adopting digital feedback technology in routine airstacking practices. Recently, advanced techniques have been developed to visualize chest wall movements and assess lung gas distribution, offering deeper insights into respiratory mechanics [9,20,21]. Combining these novel imaging methods with portable digital manometers could provide a more comprehensive and precise approach to optimizing airstacking.

Overall, this study demonstrates the potential benefits of integrating digital pressure feedback into airstacking practices, not only for enhancing caregiver performance but also for improving their physical well-being and satisfaction. These findings highlight the importance of objective tools in standardizing pulmonary rehabilitation techniques for patients with neuromuscular disorders.

Several limitations should be considered when interpreting these results. First, the inclusion of patients already familiar with airstacking may have minimized the observable impact of digital feedback on pulmonary function outcomes. Additionally, while all the caregivers received standardized training before the study, individual variations in adherence to airstacking protocols throughout the study period were not monitored, which may have influenced outcomes. Second, the 1-year study period may not have been sufficient to detect long-term changes in pulmonary function, particularly for the patients already performing airstacking. Third, while outcome assessors were blinded to group allocation, participant blinding was not feasible due to the nature of the intervention. Since the participants and caregivers were aware of whether they received digital pressure feedback, this may have influenced subjective outcome measures such as caregiver satisfaction and pain perception. Lastly, the relatively small sample size may have limited the statistical power to detect subtle differences between groups.

Future studies should include patients new to airstacking to better evaluate the full impact of digital feedback during initial training and long-term usage. Additionally, extending the study duration and increasing the sample size could provide more robust evidence of the clinical benefits of digital feedback devices. Stratifying patients by the underlying disease may help identify disease-specific responses to airstacking and digital feedback. Exploring other caregiver outcomes, such as psychological stress or quality of life, could also yield valuable insights into optimizing caregiver-patient dynamics.

## 5. Conclusions

In conclusion, while digital pressure feedback may not have significantly impacted respiratory outcomes over the study period, its clear benefits for caregivers, including reduced musculoskeletal pain and high satisfaction, underscore its value as a practical and supportive tool.

## Figures and Tables

**Table 1:** Changes in forced vital capacity (FVC%) across visits in each group.

Group	Visit 1	Visit 2	Visit 3	Visit 4	*p*-Value
1	12.69 ± 7.94	14.1 ± 10.66	11.91 ± 7.78	10.77 ± 7.93	0.164
2	21.95 ± 12.81	22.29 ± 12.05	21.4 ± 11.5	22.37 ± 11.64	
3	21.95 ± 11.5	20.56 ± 11.13	19.02 ± 10.26	20.69 ± 10.64	

Forced vital capacity expressed as a percentage of the predicted normal value (FVC%). All the values are presented as mean ± standard deviation and expressed in percentage (%).

**Table 2:** Changes in maximum insufflation capacity across visits in each group.

Group	Visit 1	Visit 2	Visit 3	Visit 4	*p*-Value
1	1461.82 ± 399.55	1360.91 ± 469.24	1384.55 ± 418.22	1451.82 ± 393.26	0.218
2	1790.00 ± 333.52	1818.33 ± 356.11	1768.33 ± 340.80	1840.83 ± 375.99	
3	1679.17 ± 640.99	1647.50 ± 630.77	1556.67 ± 704.03	1487.50 ± 587.17	

All the values are presented as mean ± standard deviation, expressed in milliliters (mL).

**Table 3:** Changes in assisted peak cough flow across visits in each group.

Group	Visit 1	Visit 2	Visit 3	Visit 4	*p*-Value
1	163.64 ± 60.05	181.82 ± 57.93	155.00 ± 41.89	161.36 ± 57.80	0.787
2	220.83 ± 45.62	232.50 ± 61.81	218.33 ± 50.42	228.75 ± 66.06	
3	208.33 ± 78.61	215.83 ± 67.35	211.25 ± 59.93	220.83 ± 69.86	

All the values are presented as mean ± standard deviation, expressed in milliliters per minute (mL/min).

## Data Availability

The original contributions presented in the study are included in the article, further inquiries can be directed to the corresponding author.

## Abbreviations

The following abbreviations are used in this manuscript:

FVC	forced vital capacity
FVC%	FVC expressed as a percentage of the predicted normal value
MIC	maximum insufflation capacity
aPCF	assisted peak cough flow
SD	standard deviation
VAS	visual analog scale
